# Blocking RAGE expression after injury reduces inflammation in mouse model of acute lung injury

**DOI:** 10.1186/s12931-023-02324-6

**Published:** 2023-01-20

**Authors:** Lynne L. Johnson, Yared Tekabe, Tina Zelonina, Xinran Ma, Geping Zhang, Monica Goldklang, Jeanine D’Armiento

**Affiliations:** Departments of Medicine, Anesthesiology, and Pathology, Columbia University, 622 West 168th St, PH 10-203, New York, NY 10032 USA

**Keywords:** Acute lung injury, RAGE, Mice, Treatment, Antibodies

## Abstract

**Background:**

Receptor for Advanced Glycated Endproducts (RAGE) plays a major role in the inflammatory response to infectious and toxin induced acute lung injury. We tested the hypothesis that a RAGE blocking antibody when administered after the onset of injury can reduce lung inflammation compared to control antibody.

**Methods:**

Male and female C57BL/6 (WT) mice were used. Forty-six received lipopolysaccharide (LPS) and 26 PBS by nasal instillation on day one, repeated on day three. On day 2, 36 mice receiving LPS were divided into two groups of 18, one treated with 200 μg of non-immune isotype control IgG and the second group treated with 200 μg of anti-RAGE Ab, each dose divided between IV and IP. Ten of the 46 were not treated. On day 4, before euthanasia, mice were injected with fluorescein isothiocyanate (FITC) labelled albumen. BALF and serum samples were collected as well as lung tissue for immunohistochemistry (IHC). BALF was analyzed for cell (leukocyte) counts, for FITC BALF/serum ratios indicating pulmonary vascular leak, and for cytokines/chemokines using bead based multiplex assays. Quantitative IHC was performed for MPO and RAGE.

**Results:**

Ten LPS mice showed minimal inflammation by all measures indicating poor delivery of LPS and were excluded from analysis leaving n = 11 in the LPS + IgG group and n = 12 in the LPS + anti-RAGE group. BALF cell counts were low in the PBS administered mice (4.9 ± 2.1 × 10^5^/ml) and high in the LPS injured untreated mice (109 ± 34) and in the LPS + IgG mice (91 ± 54) while in comparison, LPS + anti-RAGE ab mice counts were significantly lower (51.3 ± 18 vs. LPS + IgG, P = 0.03). The BALF/serum FITC ratios were lower for the LPS + anti-RAGE mice than for the LPS + IgG mice indicating less capillary leakiness. Quantitative IHC RAGE staining was lower in the LPS + anti-RAGE ab mice than in the LPS + IgG treated mice (P = 0.02).

**Conclusions:**

These results describe a four-day LPS protocol to sustain lung injury and allow for treatment and suggests that treatment aimed at blocking RAGE when given after onset of injury can reduce lung inflammation.

## Background

Receptor for Advanced Glycated Endproducts (RAGE) is a multiligand receptor found on bronchiolar epithelium, type II alveolar pneumocytes, and alveolar macrophages [1]. In response to infectious agents including influenza and pneumonia, inflammatory ligands including Acute Glycation Endproducts (AGEs), S100 calgranulins, and high mobility group box 1 (HMGB1) bind RAGE. This binding activates downstream pathways mediating pathogen-associated molecular patterns (PAMPS) and Damage Associated Molecular Patterns (DAMPS) that perpetuate and amplify the inflammatory response triggering the acute lung injury (ALI) and the acute respiratory distress syndrome (ARDS) [2–4]. Levels of RAGE ligands are elevated in the sera of patients with viral pneumonia including Covid-19 with ARDS and associated with poor outcome [5–9]. There is a published report investigating the efficacy of a RAGE blocking antibody to reduce lung inflammation in a mouse model of acute lung injury [10]. Injury was induced by acid inhalation and the antibody was administered before the injury. The results showed that both an anti-RAGE antibody and soluble sRAGE, the cleaved extracellular domain of the receptor acting as decoy, reduced lung injury. Since the treatment was given before injury, results are not directly applicable to the clinical situation where the treatment is administered after the lung injury is established and is on-going. To allow drug administration after onset of injury we altered the standard single dose LPS injury model to include a window for treatment to allow treatment after the onset of injury.

In our revised LPS administration protocol we administered LPS via nasal instillation to mice on day one and day three, treated on day two and survived mice to day four. This allowed us to compare treatment regimens in injured mice to matched control mice given PBS nasal instillation and follow survival, weights, and quantify the lung injury and capillary leakiness.

## Methods

### Animals

Procedures involving the use of animals was approved by the Institutional Animal Care and Use Committee (Columbia University). For all experiments, 6–8-week-old male and female C57/BL6 mice were obtained from Jackson Laboratories (Ellsworth, ME). The following groups of mice were used: C57BL/6 (WT) mice with LPS injury alone (n = 10) and injury control (PBS) (n = 8), WT LPS mice treated with anti-RAGE ab (n = 18) with 6 PBS controls, WT LPS treated with non-immune isotype matched control IgG (n = 18) with 12 PBS controls. Mice were weighed daily.

### LPS administration

Escherichia coli O111:B4-derived LPS (Sigma Chemical) was dissolved in sterile, endotoxin-free PBS (GIBCO) under sterile conditions. Mice are anesthetized with isoflurane, and LPS (1.5 mg/kg) is delivered via nasal instillation using a micropipette. LPS doses were repeated at day 3 and mice sacrificed on day 4. PBS was administered by nasal instillation in matching mice in treatment groups.

### Antibodies

The anti-RAGE antibody was initially produced using an immunizing peptide that contained a unique AA sequence on the V domain of the extracellular portion of the antibody. Murine monoclonal antibodies were produced by hybridoma technology and subsequently the ab was humanized and the humanized IgG1 isotype produced using the chimeric antibody intermediary process. The antibody cDNA was cloned and expressed on mammalian cells (ExpiCHO-S) and then large-scale antibody produced using plasmid technology (Fair Journey Biologics, Porto Portugal). Testing with this antibody revealed the following. By FACS analysis the antibody bound to cells from several RAGE-expressing cell lines. It significantly reduced AKT phosphorylation induced by S100B in aortic smooth muscle cells in dose responsive manner, and inhibited S100B -induced TNFα and IL-1b secretion in dose responsive manner. It inhibited HMGB1- induced IL-6 secretion from human peripheral bone marrow cells. The peptide sequences of this antibody are the same as for the antibody referred to as CR-3 in our recent publications [11, 12].

### Drug administration

Animals were allowed to recover for 24 h following injury before administration of any drug treatment. Dose of either anti-RAGE ab or isotype control non-immune IgG (200 µg) were divided between IV administration and IP (volume 200 µl per divided dose). Animals are closely monitored for changes in appetite, weight loss, and respiratory difficulty.

### Euthanasia

At day 4 (24 h after last LPS dose) mice were euthanized with isoflurane followed by cervical dislocation. Two hours before euthanasia, mice were injected with FITC-labeled albumin (Sigma-Aldrich, no. A9771) to assess potential pulmonary vascular leak. Lungs, serum, and BALF (Bronchoalveolar lavage fluid) are taken for analysis. Briefly, the chest was opened, the right ventricle cannulated and about 250 μL of blood removed. BALF was removed and the right lung snap-frozen for later analysis. Blood sample was taken and centrifuged to separate and collect serum for frozen storage. The left lung was preserved in 10% formalin for paraffin embedding, sectioning, and subsequent staining.

### BALF analysis

BALF samples were centrifuged (Cytospin 4 Thermo Scientific) and the supernatant frozen for cytokine analysis. The cell pellets were placed in cell counting instrument (Countess, Invitrogen Thermo fissure Scientific) to count living cells. Slides were made of the centrifuged cells, stained with DifQuik (Polyscience) and examined under microscope to confirm that they were neutrophils. Results were expressed as cells × 10^5^/ml.

Samples of serum and BALF were loaded into well plates and fluorescence intensity readings taken using a microplate reader (Spectrum Max iD3, Molecular Devices).

### Immunohistochemistry

Paraffin embedded lungs were sectioned into 5-micron slices and mounted on slides. Serial tissue sections were stained for hematoxylin and eosin (H&E) and for myeloperoxidase (MPO) (rabbit anti-myeloperoxidase antibody, 1:8000, abcam) and for RAGE (human anti-RAGE antibody, 50 µg/ml). Secondary staining was performed with HRP-conjugated goat anti-rabbit or goat anti-human antibody (1:200). Staining for MPO and for RAGE (brown staining) was quantified by IHC on five sections from each lung section using Image Pro Plus software (Media Cybernetics, Silver Spring, MD).

### Dual fluorescence

To determine the cell type expressing RAGE in lung inflammation, dual fluorescent confocal microscopy studies were performed. Briefly, lung sections (5-μm-thick) were stained for MPO and RAGE with the respective antibodies as described above. The slides were then co-stained by incubating with fluorescent tagged secondary antibodies (Texas Red and fluorescein isothiocyanate) (manufacturers). The images were examined using Nikon Eclipse 50i confocal fluorescence microscope (Nikon, Melville, NY, USA) and Image Pro Plus software (Diagnostic Instruments, Inc., Sterling Heights, MI, USA).

### Cytokine analysis

Frozen BALF samples from all experiments included in the final data were sent to EVE technologies (Calgary, Alberta, CA) for bead based multiplex assays using Luminex technology. From the EVE technologies 32 plex mouse panel analysis, 12 cytokine/chemokines were selected for comparative analysis.

### Statistical analysis

The following variables were compared between groups: vascular leakiness, inflammatory cell counts in BALF, lung MPO and RAGE staining on IHC. Lung MPO values were plotted vs. BALF inflammatory cell counts. An unpaired, two-tailed Student’s t test was used for comparisons between experimental groups. One-way analysis of variance was used for statistical analysis of three or more groups followed by the Bonferroni post hoc test. All statistical analysis was performed using GraphPad Prism software (GraphPad Software, La Jolla, CA).

## Results

All animal experiments were performed with the approval of the Institutional Animal Care and Use Committee of Columbia University. C57BL/6 mice (equal numbers of male and female) were obtained from Jackson Laboratories (Bar Harbor, ME).

### Animals

All mice except one survived to the end of the experiments and one additional mouse died on the final day. For the C57BL/6 vs. PBS treated experiments the average first dose of LPS was 32.5 ± 6.9 mg and second dose 29.0 ± 5.4 mg. The LPS treated mice lost 5.15 ± 2.03 g and the PBS mice gained 0.74 ± 0.84 g. For the blinded dosing experiments the IgG treated mice received an average first dose of LPS of 34.4 ± 5.0 mg and 30.3 ± 5.9 s dose. The anti-RAGE ab mice received 37.4 ± 5.8 mg LPS for first dose and 32.6 ± 7.4 for second dose. Weight changes for each of the four groups were the following. For LPS without treatment: − 5.15 ± 2.03 g, for the PBS nasal instillation: + 0.74 ± 0.84 g, for LPS + IgG: − 4.2 ± 1.3 g, for the LPS + anti-RAGE mAb: − 3.9 ± 1.7 g.

Six of the LPS injured mice treated with non-immune IgG and four of the LPS mice treated with anti-RAGE ab did not show inflammation by BALF cell count or on whole lung H&E histology and were determined to have had poor delivery of LPS to the lungs. These mice were excluded from analysis leaving 11 mice in the non-immune IgG treated group and 12 mice in the anti-RAGE ab treated group. Prior work done in Dr D’Armiento’s laboratory has shown that some mice do not receive the full LPS doses due to upper respiratory blockage, poor inspiration, or airway resistance to the toxin. Because of the longer protocol (4 days vs. 2 days) to allow dosing, we could not identify these experiments until the end of the protocol.

### Lung inflammation

**Fig. 1:**
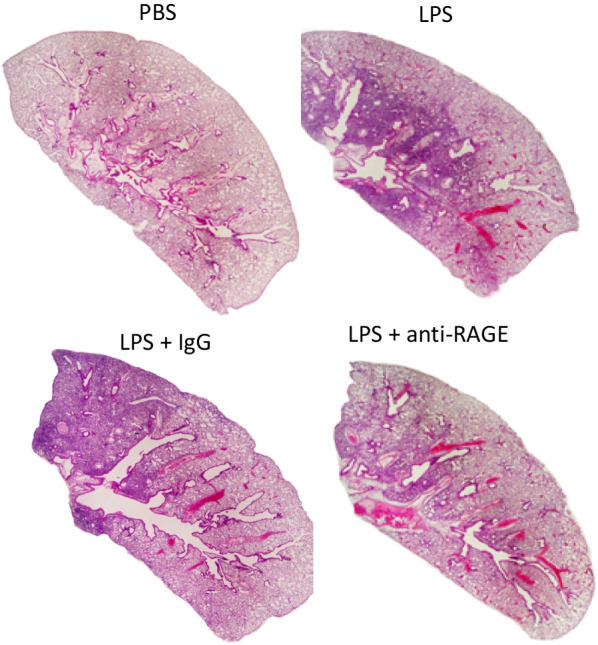
H&E staining. The whole lung section from mouse receiving PBS nasal instillation shows normal lung architecture. The lung from mouse receiving LPS nasal instillation in contrast shows intense and consolidated centro-lobular infiltration of inflammatory cells. The lung from the LPS injured mouse treated with non-immune IgG also shows dense staining indicating extensive infiltrates. The section from the LPS injured mouse treated with anti-RAGE ab shows visually less inflammatory infiltrates

The gross degree and extent of lung inflammation was assessed visually on lung histology from representative experiments as shown in Fig. 1. These images represent whole lung displays of lung sections stained with H&E and stitched together from typical examples of mice with PBS inhalation, mice with LPS instillation without antibody treatment, and mice with LPS injury treated with IgG or with anti-RAGE ab. As shown by the purple staining there is intense infiltration of cells (predominantly neutrophils) in the LPS injured and little visual difference in extent of injury between LPS alone and LPS with IgG treatment while the lung sections from mice treated with anti-RAGE ab show lesser extent of cell infiltration.

### BALF cell counts and FITC ratios

**Fig. 2:**
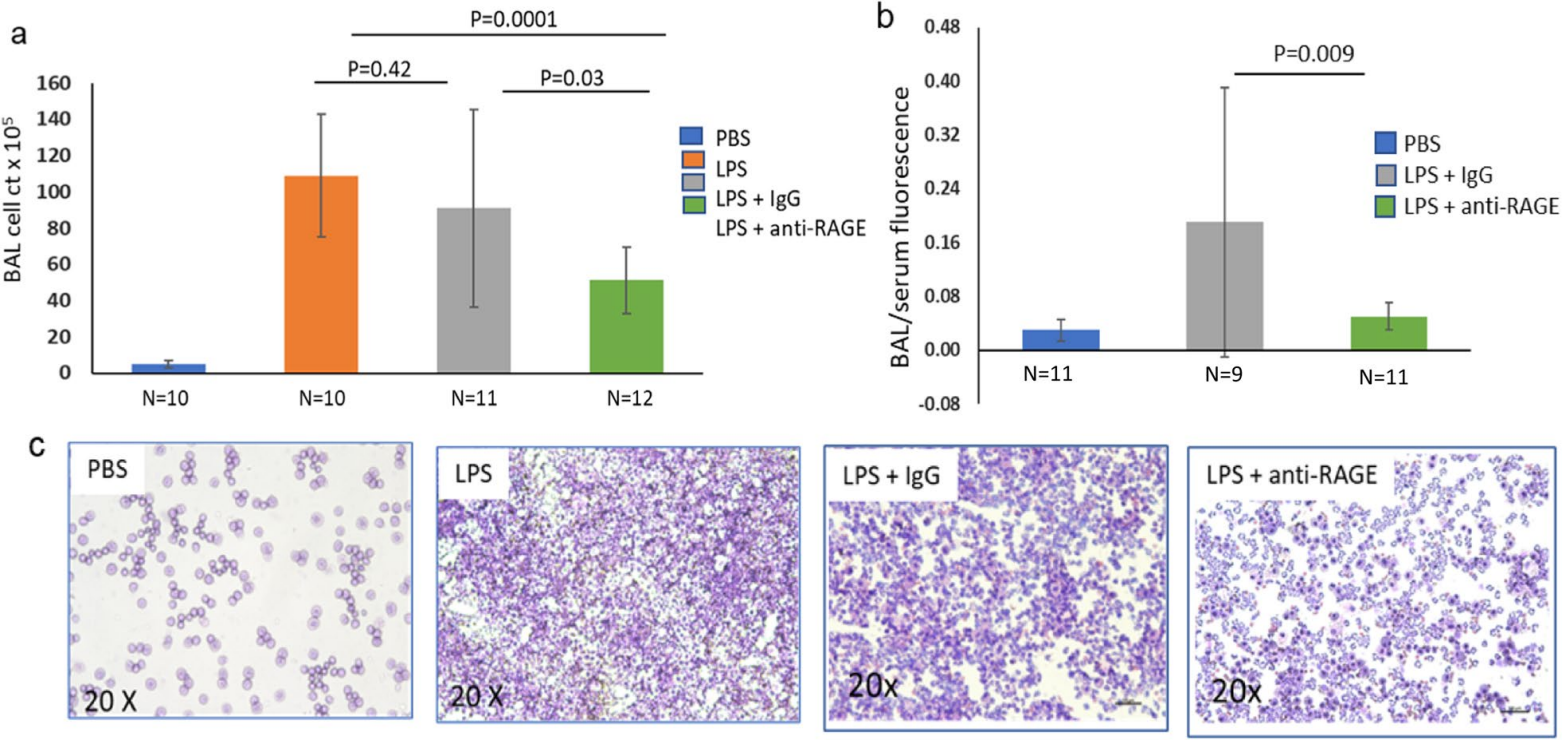
BALF cell count: Panel **a** shows bar graph of BALF cell count × 10^5^/ml for the four groups with significance levels for statistical differences between groups. See text. Panel **b** shows bar graphs for ratios of fluorescence intensity readings taken using a microplate reader for the PBS, LPS + IgG and LPS + anti-RAGE ab groups. Panel **c** shows H&E staining of the BALF samples for the 4 groups

Lung inflammation was quantified from neutrophil cell counts of the BALF from mice in each of the four groups as described in the Methods section. Mice with low inflammatory cell counts in the LPS groups were eliminated from group analysis as described above. In addition to the cell counts for the four groups we also measured the ratio of fluorescence in the BALF to the serum fluorescence following IV injection of fluorescein isothiocyanate (FITC). This ratio is an index of pulmonary capillary leakiness caused by the inflammatory damage. The results of the BALF cell counts and FITC ratios for three groups are shown in Fig. 2. Data were insufficient for FITC ratios for the WT LPS injured (no treatment) experiments done early in the project due to malfunctioning reader which was replaced by well-functioning reader for all subsequent experiments.

As shown in Fig. 2 the mean value ± standard deviation for BALF cell count for the PBS inhalation group was low (5.0 ± 2.1 cells × 10^5^/ml). The photo taken of the slide of centrifuged cells shows sparse scattered cells (neutrophils). For the LPS injured mice without treatment the mean value for BALF cell count was high (109 ± 34 cells × 10^5^/ml) and the photo of cells shows packed neutrophils. The BALF from mice with LPS plus IgG was high (91.0 ± 54.4 cells × 10^5^/ml), and photo of the centrifuged cells looks similar to the untreated group (P = 0.42). The BALF from the mice receiving anti-RAGE ab had lower neutrophil counts (51.3 ± 18.3) than the IgG treated mice (P = 0.03) and the photo of the centrifuged BALF shows less numerous cells. The FITC ratios of BAL/serum are shown in panel b in Fig. 2. The significantly lower values in the LPS + anti-RAGE dab group indicates less capillary leakiness than for the LPS + IgG group.

### MPO staining

**Fig. 3:**
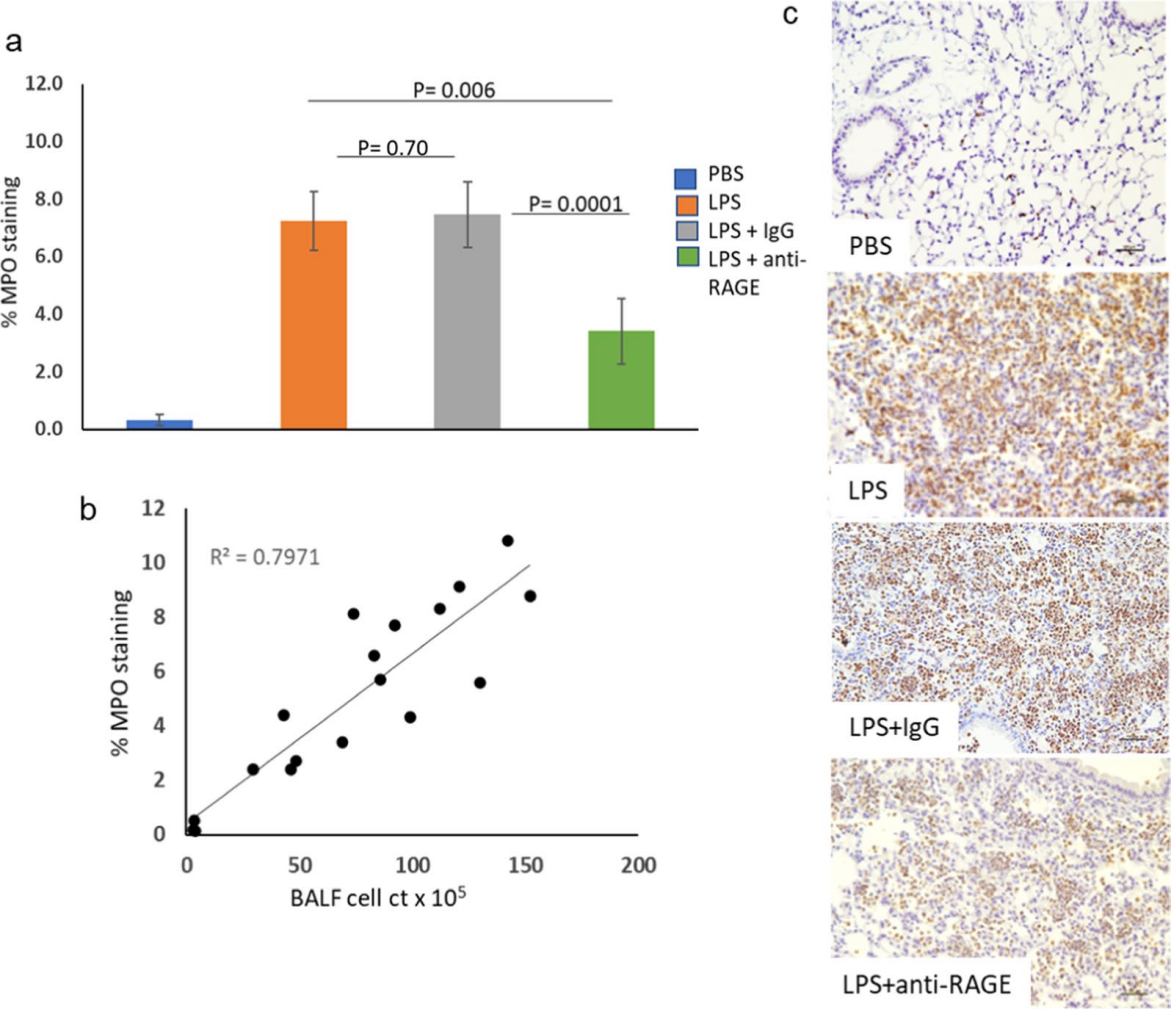
IHC staining for MPO: Panel **a** shows bar graph of average for sections from 6 experiments for each group stained for MPO as average % areas over all sections for each lung. Panel **b** shows graph of significant correlation between %MPO staining and BAL cell count × 10^5^/ml. Panel **c** shows the stained tissue sections of areas of most intense infiltration from representative experiments from each group. The brown chromogen identifies MPO positive cells

Myeloperoxidase (MPO) is a leukocyte-derived peroxidase enzyme that is abundantly expressed in neutrophil granulocytes and contributes to tissue damage during inflammation. Tissue staining for MPO was performed on lung tissue sections from all four experimental groups. As shown in Fig. 3 MPO staining as % staining measured from 200X sections for 6 representative experiments from each group was very low in the PBS inhalation experiments (0.32 ± 0.19) and high for the LPS alone and LPS plus IgG experiments (7.74 ± 2.66 and 7.25 ± 1.02) and lower for the LPS plus anti-RAGE ab experiments (3.41 ± 1.13), which was significantly lower than for the LPS alone and LPS plus IgG groups. When the % staining for MPO for all individual experiments for the 4 groups are plotted vs. BALF cell count cells × 10^5^/ml the correlation was highly significant with R^2^ = 0.7971 and P < 0.0001.

### Cytokine analysis

The 32-sample mouse cytokine/chemokine panel analysis was performed by EVE technologies (Alberta, Canada) on BALF samples from all experiments included in analysis. Eleven of the cytokines/chemokines measured were relevant to lung inflammation. These 11 include granulocyte colony stimulating factor (G-CSF), IL-1β, IL-6, C-X-C motif chemokine (IP-10), keratinocyte-derived chemokine (KC), monocyte chemotactic protein 1 (MCP-1), macrophage inflammatory proteins -1α and -1β, regulated upon activation normal T cell expressed and presumably secreted chemokine (RANTES), and tumor necrosis factor (TNFα). The results are summarized in the Table. Measurements are expressed as concentrations in pg/ml.

**Table:** 

	G-CSF	IL-1β	IL-6	IP-10	KC	MCP-1	MIG	MIP-1α	MIP-1β	RANTES	TNFα
PBS, n = 11											
Mean	7.45	2.21	3.16	3.16	6.70	9.23	0.67	33.39	25.07	0.83	1.61
SD	3.83	0.60	3.23	1.52	2.10	2.74	0.43	13.16	4.27	0.76	1.62
LPS, n = 10											
Mean	5753	167	8392	2788	242	546	6692	904	2204	205	206
SD	2446	91	3761	1016	80	365	2064	407	1552	138	70
IgG, n = 12											
Mean	5640	284	5887	3232	385	880	5109	1172	3569	199	245
SD	2334	164	4119	2231	247	856	2227	546	3029	111	98
CR-3, n = 9											
Mean	4225	92	5354	2373	185	543	3080	815	2357	91	225
SD	2398	69	4248	1970	89	508	2056	348	1655	52	161
P values											
IgG vs. LPS	0.92	0.105	0.24	0.19	0.16	0.34	0.13	0.25	0.26	0.92	0.35
anti-RAGE vs. LPS	0.21	0.04	0.29	0.14	0.21	0.99	0.003	0.63	0.85	0.04	0.76
anti-RAGE vs IgG	0.19	0.007	0.77	0.78	0.034	0.31	0.05	0.10	0.29	0.02	0.73

Mean values for all the cytokines/chemokines measured were low in the PBS inhalation mice. For three proteins (IL-1β, KC, and RANTES), mean sample values for the anti-RAGE ab treated mice were significantly lower than for IgG treated and for one (MIG) the P was borderline significant. The highest significance was for IL-1β which is a key moderator of the initiation of the inflammatory response in lung disease. There was a concordance in significance values between the anti-RAGE ab treated group vs. non-immune IgG treated mice and between the anti-RAGE ab treated group vs. LPS untreated mice for IL-1β, MIG, and RANTES, but not for KC.

### RAGE expression

**Fig. 4:**
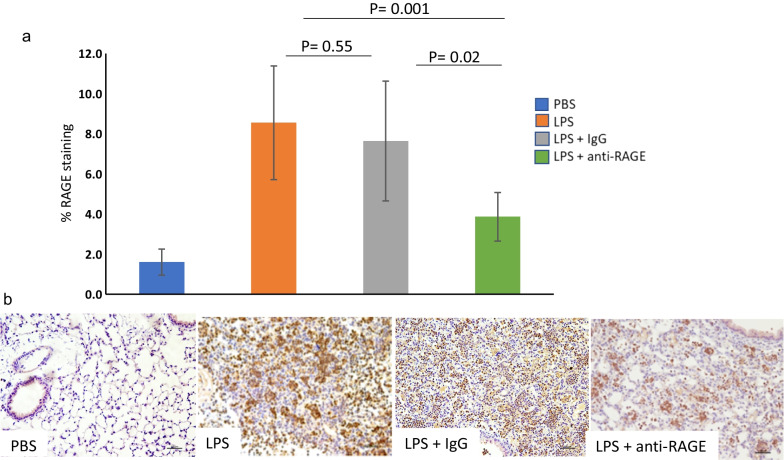
IHC staining for RAGE: Bar graph on panel **a** shows values for % RAGE staining for the four groups. The P values show significantly less RAGE staining for lung sections from the LPS + anti-RAGE ab compared to the LPS + IgG mice. Panel **b** shows representative IHC stained lung sections from each of the 4 groups. The brown chromogen indicates more intense RAGE staining in the LPS mice and the LPS + IgG mice compared to the LPS + anti-RAGE mice

RAGE expression by IHC as average % staining per 200X fields was low in the mice with PBS inhalation (1.6 ± 0.65%). The average % staining in the LPS injured mice was 8.55 ± 2.84% which was not different from the mice injured with LPS and treated with IgG (7.63 ± 2.99%, P = 0.54). For mice treated with anti-RAGE ab, RAGE staining was lower (3.86 ± 1.21) compared to LPS (P = 0.001) and compared to IgG treated mice (P = 0.017). The stained section (20 X) displayed in the bottom panel of Fig. 4 show RAGE staining (brown chromogen). The most intense staining is in the section from an LPS injured mouse. While there is brown staining in the section from the mouse injured with LPS and treated with anti-RAGE ab it is visually less than in the section from the mouse injured with LPS and treated with IgG. Similar to results from the MPO stained sections, regions selected are from the areas of lung showing the greatest inflammation. The overall % is the average of 5 areas of the lung representing the entire lung field (Mag. X200).

### Dual fluorescence staining

**Fig. 5:**
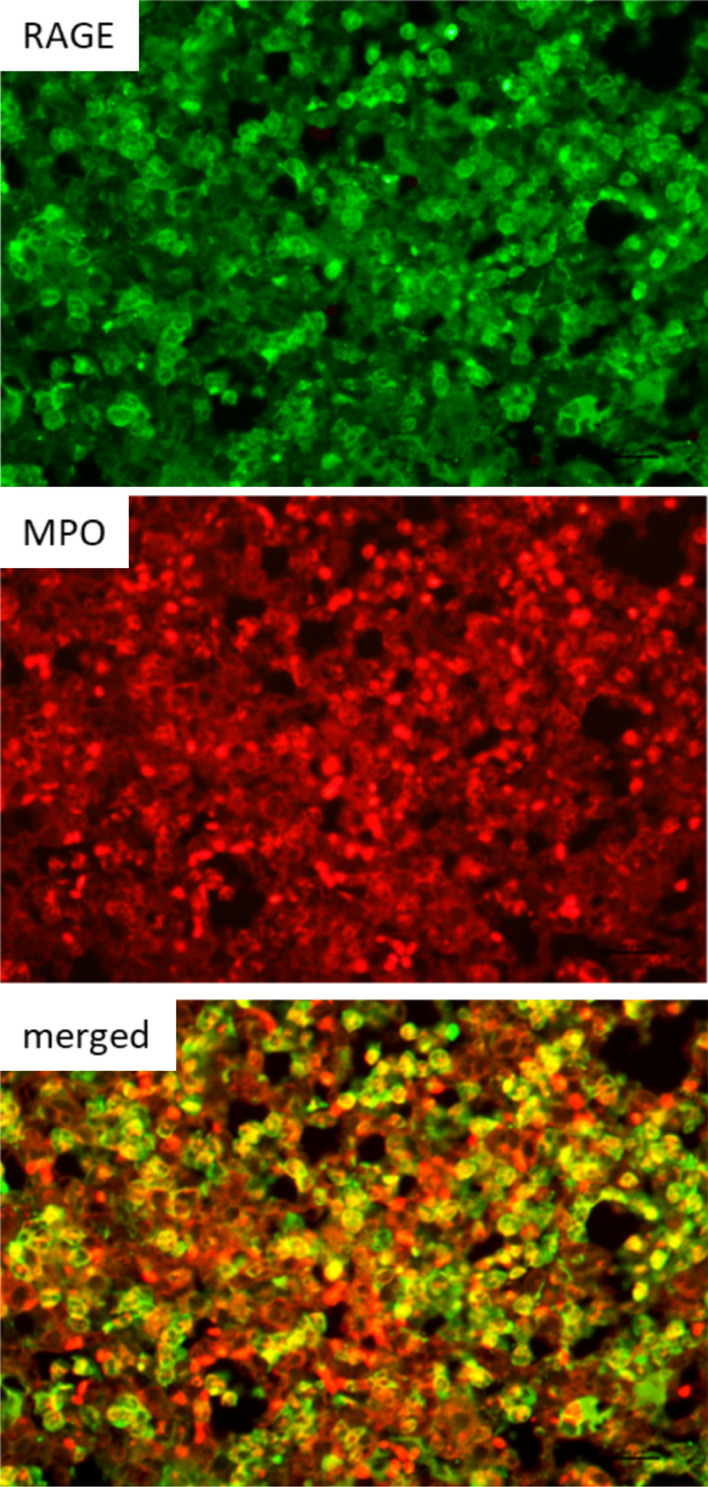
Dual fluorescence: Immunofluorescence staining for MPO (Texas red) and RAGE (indocyanine green) In merged sections the yellow color represents cells that co-localize both stains- leukocytes expressing RAGE

Serial lung sections from an LPS injured mouse stained for MPO and RAGE were counterstained with fluorescent labels (Texas red for MPO and fluorescein for RAGE) and shown in Fig. 5 below. Sections are merged and cells that co-localized both fluorescence stains were yellow. Slide 5 shows the extent of co-localized cells and suggests that neutrophils which are the most abundant cell in this lung injury model are expressing RAGE.

## Discussion

Acute Respiratory Distress Syndrome (ARDS) results from diverse injuries including viral, bacterial, toxic, or trauma. Despite these diverse causes the lung injury has common pathological features including cytokine release which promotes increased lung permeability and leukocyte infiltration into the alveolar space manifest by clinical picture of increasing respiratory distress and hypoxemia and acute respiratory failure. On histopathology there is diffuse alveolar damage (DAD). Prominent among the several known molecular pathways implicated in the instigation and perpetuation of acute lung injury is receptor for advanced glycation endproducts (RAGE). RAGE is a multi-ligand receptor expressed on cells throughout the body including in the lung where it is found in bronchiolar epithelia, type II alveolar pneumocytes, and alveolar macrophages as well as the endothelium of larger arteries [1, 14]. The full receptor consists of 5 domains: the cytosolic domain, which is responsible for signal transduction, the transmembrane domain which anchors the receptor in the cell membrane, the variable domain (V) which binds the RAGE ligands, and two constant (C) domains [16]. Cleavage of the extracellular domain releases soluble RAGE (sRAGE) into the circulation. Binding of ligands to the cell bound receptor activates down steam pathways that activate inflammatory pathways. Increases in receptor and ligands leads to further increases in levels of receptor and ligands in a positive feedback loop [13]. An antibody that binds to the V domain of RAGE, blocks the attachment of ligands to the receptor which in turn leads to reduced receptors and reduced ligands. This is the presumed mechanism for efficacy of our anti-RAGE Ab in reducing inflammation in ARDS. Among the many ligands identified to bind RAGE include advanced glycation endproducts (AGEs), S100 calgranulins, and high mobility box protein (HMGB1). These ligands in addition to sRAGE have been found in serum samples from patients with bacterial, and viral (including Covid-19) pneumonia with ARDS and are associated with poorer outcomes [5–7].

Because of the critical role played by RAGE in diseases causing progressive respiratory failure, developing an approach to reduce RAGE expression in lung injury is an important endeavor. There is one published report showing beneficial effect of blocking RAGE expression in a mouse lung injury model [10]. In this study investigators used an ALI mouse model created by instilling hydrochloric acid via orotracheal route and by administering either an anti-RAGE mAb or sRAGE (as decoy) prior to the injury and found reduced lung injury, improved arterial oxygenation, and decreased alveolar inflammation [10]. They gave treatment before injury. In the clinical situation, treatment is not begun until after the symptoms have developed. We therefore developed a protocol to allow us to begin treatment after the injury.

The endotoxin lipopolysaccharide (LPS) a glucosamine-based phospholipid that makes up the outer monolayer membrane of most gram-negative bacteria is used to induce acute lung injury (ALI) in mice to study pathways of inflammation and immunity [15, 16]. LPS acts as the prototypical endotoxin because it binds the CD14/TLR4/MD2 receptor complex in many cell types, but especially in monocytes, dendritic cells, macrophages and B cells, which promote the secretion of pro-inflammatory cytokines, nitric oxide, and eicosanoids [15]. The mouse model of ALI does not accurately reproduce all the histological features of DAD in humans however the LPS induced ALI model is commonly used because it does recapitulate the main features of ALI including histological evidence of tissue injury, alteration of the alveolar capillary barrier, inflammation, and physiological dysfunction [17]. According to the ATS workshop on experimental model of ALI in animals, any three of these main criteria must be met. In the study we report, endpoints measured and reported include three of these criteria: lung histology, FITC-albumen measurements of capillary leak, and inflammatory cell counts in the BALF [18]. Our findings showed improvements in each of the above criteria in the mice treated with anti-RAGE mAb compared to those treated with non-immune IgG.

From the panel of cytokines/chemokines we had run on BALF samples, 3/11 assays showed P values that were significant for differences between the mice treated with anti-RAGE ab and with non-immune IgG and one assay was borderline significant at P = 0.05. These four were IL-1β cytokine, KC (CXC) or keratinocyte chemoattractant chemokine, MIG or monokine induced by gamma interferon chemokine (CXCL9), RANTES (regulated upon activation, normal T cell expressed and secreted) (CCL5) chemokine. Chemokines or chemotactic cytokines are small cytokines secreted by cells that induce directional movement of leukocytes. Lung injury by bacteria, viruses, or toxins leads to excessive recruitment of neutrophils to the lung with resultant inflammatory injury to the alveolo-capillary membrane which leads to increased permeability as shown by BAL/serum FITC ratios, and accumulation of fluid in the airspaces [19]. Resident lung tissue cells, leukocytes, and cytokine-activated endothelial and epithelial cells secrete chemokines which attract and trap neutrocytes in the alveolar fluid space as shown by the BALF cell counts. High levels of lung chemokines recruit macrophages in ALI [19]. IL-1β is released by activated alveolar macrophages and this cytokine further stimulates other lung cells to produce more CC and CXC chemokines, recruiting more neutrophils and perpetuating the cycle of inflammation.

Patients with ARDS show elevated levels of sRAGE in bronchial fluid and serum. In addition to its role in stimulating inflammatory pathways, RAGE may regulate lung fluid balance in direct response to injury. One group of investigators found that alveolar fluid clearance was higher and pulmonary vascular albumen leakage reduced in RAGE^−/−^ mice compared to wild type following in LPS following LPS injury [20]. Similar findings were reported following treatment with anti-RAGE ab in acid lung injury mouse model of ALI [10]. These investigators hypothesized that blocking RAGE may reduce alveolar type 1 epithelial cell injury which would reduce chemokine release and help restore alveolar clearance [10, 20]. Our data show similar results when a RAGE blocking antibody is administered after onset of injury and therefore may be more relevant to extrapolate to potential therapeutic usefulness in clinical disease.

## Conclusions

We describe a protocol for producing a model of on-going acute lung injury allowing first dose of treatment to be administered after the onset of injury which is more applicable a model for testing a drug administered in a clinical situation. The large amount of published data show that RAGE is activated in ARDS in the setting of lung infection or in systemic sepsis from other causes, all of which point to the potential value of administering a RAGE blocking antibody to ameliorate lung damage in these patients.

## Data Availability

All data and material will be provided to interested researchers upon request.

## Abbreviations

AGEs: Advanced Glycated Endproducts
AKT: Protein kinase B
ALI: Acute lung injury
ARDS: Acute respiratory distress syndrome
BALF: Bronchoalveolar lavage fluid
FACS: Fluorescence activated cell sorting
FITC: Fluorescein isothiocyanate labelled albumen
HMGB1: High mobility group box
IHC: Immunohistochemistry
LPS: Lipopolysaccharide
MPO: Myeloperoxidase
PBS: Phosphate buffered saline
RAGE: Receptor for Advanced Glycated Endproducts
